# Toxicity, physiological response, and biosorption mechanism of *Dunaliella salina* to copper, lead, and cadmium

**DOI:** 10.3389/fmicb.2024.1374275

**Published:** 2024-03-28

**Authors:** Mingze Gao, Na Ling, Haiyan Tian, Chunqiu Guo, Qiyao Wang

**Affiliations:** ^1^Pharmaceutical Engineering Technology Research Center, Harbin University of Commerce, Harbin, China; ^2^Engineering Research Center for Natural Antitumor Drugs, Ministry of Education, Harbin, China; ^3^School of Pharmaceutical Sciences, Shandong University, Jinan, China

**Keywords:** *Dunaliella salina*, heavy metals, physiological response, antioxidant, biosorption

## Abstract

**Background:**

Heavy metal pollution has become a global problem, which urgently needed to be solved owing to its severe threat to water ecosystems and human health. Thus, the exploration and development of a simple, cost-effective and environmental-friendly technique to remove metal elements from contaminated water is of great importance. Algae are a kind of photosynthetic autotroph and exhibit excellent bioadsorption capacities, making them suitable for wastewater treatment.

**Methods:**

The effects of heavy metals (copper, lead and cadmium) on the growth, biomolecules accumulation, metabolic responses and antioxidant response of *Dunaliella salina* were investigated. Moreover, the Box-Behnken design (BBD) in response surface methodology (RSM) was used to optimize the biosorption capacity, and FT-IR was performed to explore the biosorption mechanism of *D. salina* on multiple heavy metals.

**Results:**

The growth of *D. salina* cells was significantly inhibited and the contents of intracellular photosynthetic pigments, polysaccharides and proteins were obviously reduced under different concentrations of Cu^2+^, Pb^2+^ and Cd^2+^, and the EC50 values were 18.14 mg/L, 160.37 mg/L and 3.32 mg/L at 72 h, respectively. Besides, the activities of antioxidant enzyme SOD and CAT in *D. salina* first increased, and then descended with increasing concentration of three metal ions, while MDA contents elevated continuously. Moreover, *D. salina* exhibited an excellent removal efficacy on three heavy metals. BBD assay revealed that the maximal removal rates for Cu^2+^, Pb^2+^, and Cd^2+^ were 88.9%, 87.2% and 72.9%, respectively under optimal adsorption conditions of pH 5-6, temperature 20-30°C, and adsorption time 6 h. Both surface biosorption and intracellular bioaccumulation mechanisms are involved in metal ions removal of *D. salina*. FT-IR spectrum exhibited the main functional groups including carboxyl (-COOH), hydroxyl (-OH), amino (-NH2), phosphate (-P=O) and sulfate (-S=O) are closely associated with the biosorption or removal of heavy metalsions.

**Discussion:**

Attributing to the brilliant biosorption capacity, *Dunaliella salina* may be developed to be an excellent adsorbent for heavy metals.

## Graphical abstract

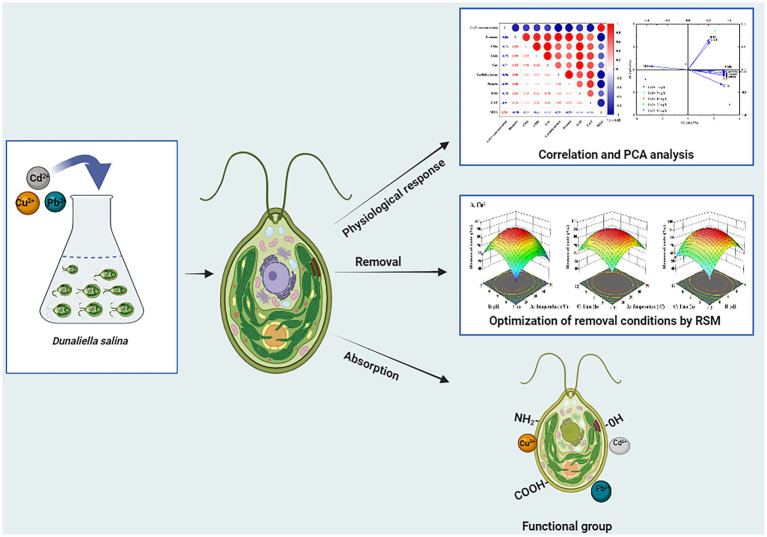

## 1 Introduction

Heavy metal pollution (such as copper, lead, cadmium, arsenic, mercury, and chromium) has become a global and the most serious environmental problem, which is generated from a variety of activities such as unreasonable mining, application of pesticides and fertilizers, manufacturing of electronics, industrial waste discharge, household garbage disposal, food processing, chemicals, pharmaceutical, aquaculture, textile, and metallurgy (Vo et al., 2020; Luo et al., 2022). Heavy metals are very difficult to biodegrade but can be accumulated through food chain and finally enter the human body, ultimately posing serious health risks to living organisms and leading to the deterioration of environment (Lu Y. et al., 2015; Ajiboye et al., 2021; Gokul et al., 2023). In light of this issue, the World Health Organization (WHO) and the US Environmental Protection Agency (USEPA) have established rigorous standards for the permissible levels of heavy metal discharge into the environment. In particular, heavy metals such as copper (Cu), lead (Pb), zinc (Zn), cadmium (Cd), chromium (Cr), and nickel (Ni) have garnered the most significant attention, and these are categorized as the priority pollutants due to their high toxicity (Cheng et al., 2019).

Copper (Cu) is an essential micronutrient for humans, plants, and animals, as well as a cofactor for chlorophyll and a variety of enzymes, such as oxidoreductases and nitrate reductase (Peers and Price, 2006; Perales-Vela et al., 2007). Cu participates in many physiological and biochemical processes through assimilating and transferring electrons, including photosynthesis, respiration, signal transduction, and the antioxidant system (Moenne et al., 2016; Maleki et al., 2017). Nevertheless, excessive copper may cause liver cirrhosis, diarrhea, vomiting, movement disorders, and perceptual nerve disorders (Coelho et al., 2020; Kahlson and Dixon, 2022). Cadmium (Cd) and lead (Pb) are two important heavy metal elements that are famous for causing global environmental pollution. Not only can they lead to the dysfunction of physiological metabolism, inhibition of photosynthesis, disruption of membrane protein, and even the death of aquatic plants (Dao and Beardall, 2016; Samadani et al., 2018) but also cause harm to the organs of human body, such as the liver, kidney, nervous system, and hematopoietic system, and even directly affect the intellectual development of children (Khanam et al., 2020).

The health risk associated with heavy metals has aroused worldwide awareness. Researchers all over the world have explored multiple techniques to remove the heavy metals from the environment. Common treatment approaches are categorized as physical, chemical, or biological, including electrodialysis, activated carbon adsorption, membrane separation, reverse osmosis, and electrochemical precipitation (Ajiboye et al., 2021; Wijeyawardana et al., 2022), but present unattractive due to high processing cost and low efficiency. Biosorption has emerged as a promising alternative to conventional methods. This process involves various physicochemical mechanisms, such as chelation, complexation, ion exchange, microprecipitation, and physical adsorption (An et al., 2019; Cheng et al., 2019). Numerous studies have sought to authenticate appropriate biosorbents that can effectively remove heavy metals. Generally, these biosorbents are derived from agricultural wastes, plant adsorbents, algae, bacteria, and fungi. By utilizing these natural materials, biosorption offers a sustainable and environmentally friendly approach to the removal of heavy metals.

Among the biosorbents, algae-based biosorbents, in particular, emerge as prime candidates for environmental remediation due to their outstanding adsorption capabilities, coupled with the negligible production of toxic byproducts, cost-effectiveness, conservation of time and energy, user-friendly handling property, ubiquitous presence, abundance, and renewability (Kiran Marella et al., 2020; Lin et al., 2020; Greeshma et al., 2022). Additionally, the residual algal biomass after biosorption can be disposed via municipal landfills or repurposed into valuable byproducts, such as biochar or biofuel. Multiple studies have investigated the effects of heavy metals on algae physiological and biochemical parameters and algal biosorption. Han et al. (2008) and Cao et al. (2015a) found copper, zinc, and lead (>50 mg/L) significantly inhibited the growth of *Cladophora*, *Ulva pertusa,* and *Ulva armoricana,* impeded the photosynthesis, and reduced the contents of chlorophyll, total soluble sugar, and protein. Similarly, Shanab et al. (2012) discovered that lower concentrations of Hg^2+^, Pb^2+^, and Cd^2+^ (5–20 mg/L) stimulated the growth of *Phormidium ambiguum*, *Pseudochlorococcum typicum,* and *Scenedesmus quadricauda* and improved the contents of chlorophyll a and protein, while higher concentrations (40–100 mg/L) hindered algal growth. Machado et al. (2015) found that the chlorophyll content, maximum quantum efficiency of photosystem II, and reduced esterase activity were all influenced in *Pseudokirchneriella subcapitata* under the stress of heavy metals (Cu^2+^, Zn^2+^, Cd^2+^, and Cr^6+^). Kováčik et al. (2017) discovered that Cd exposure could increase the biomass and accumulation of ascorbic acid, phenols, and thiols, along with oxidative stress in *Scenedesmus acutiformis*. Furthermore, Edris et al. (2014) utilized *Chlorella vulgaris* to remove Cd and Pb, with the maximum adsorption capacities of 149.9 mg/g for Cd and 178.5 mg/g for Pb. Lekshmi et al. (2022) reported that the removal efficiency was 94.8 ± 3.3%, 90.8 ± 1.4%, and 87.5 ± 2.3% for Cd, Zn, and cobalt, respectively. In summary, multiple algae exhibited excellent bioadsorption capacities, making them suitable for wastewater treatment.

*Dunaliella salina,* a halophilic and unicellular eukaryotic green alga that lacks cell wall, is extensively distributed in high salinity water. It has been widely employed in bioassays for its ease cultivation, high availability, low cost, and the ability to synthesize substantial quantities of protein, lipid, glycerol, and carotenoids under the conditions of high salinity, high/low temperature, heavy metals, and nutrient deficiency (Feng et al., 2020; El Agawany et al., 2021). Multiple literature studies have confirmed that heavy metals such as Cd, Pb, and Zn can influence the growth, accumulation of photosynthetic pigments, and physiological process of *Dunaliella*, accompanied by the changes in antioxidant enzymes (Zhu et al., 2019; Ziaei et al., 2023). Simultaneously, *Dunaliella* demonstrated excellent biosorption capacity of heavy metals, such as Pb^2+^, Zn^2+^, and Ni^+^. The maximal adsorption of Zn^2+^ was captured under the conditions of Zn^2+^ concentration, algae biomass, initial pH, and duration at 25 mg/L, 0.5 g/L, pH 7.59, and 13.72 h, respectively, by Box–Behnken design (BBD). Moreover, Fourier transform infrared spectroscopy (FT-IR) analysis indicated these functional groups such as -OH, -CH_2_, -COOH, and -P=O and may participate in the biosorption of *Dunaliella* sp. AL − 1 (Elleuch et al., 2021a).

Therefore, the purpose of this study is to scrutinize the acute toxic effects of heavy metals (Cu, Pb, and Cd) on the growth and multiple physiological parameters of *D. salina,* including the contents of photosynthetic pigments, polysaccharides, protein, and the response to heavy metal stress, such as antioxidant enzymes superoxide dismutase (SOD), catalase (CAT), and malondialdehyde (MDA). Subsequently, BBD was employed to optimize the removal efficiency of *D. salina* on multiple heavy metals, and FT-IR was performed to exploit the biosorption mechanism. These findings will offer a theoretical foundation for developing an excellent biosorbent of *D. salina* to remove heavy metals from wastewater.

## 2 Materials and methods

### 2.1 Algal culture

*Dunaliella salina* was obtained from the Key Laboratory of Marine Biological Engineering, Ningbo University. *D. salina* was maintained and cultured in a modified f/2 medium at 22–24°C, pH 7–8, light intensity 3,000–5,000 lux, and light for 12 h followed by 12 h of darkness (Ling et al., 2021). The flasks were gently shaken three times daily. All experiments were conducted in triplicate.

### 2.2 The acute toxicity of *Dunaliella salina*

Metal stock solutions of copper, lead, and cadmium were prepared by dissolving the analytical grade of copper chloride (CuCl_2_), lead chloride (PbCl_2_), and cadmium chloride hydrate (CdCl_2,_ H_2_O) in distilled water. The acute toxicity test of heavy metals on *Dunaliella salina* was performed. In brief, 1 × 10^6^ cells/ml of *D. salina* in 250 mL flasks containing 150 mL of culture in exponential phase was exposed to different concentrations of Cu^2+^ (5, 10, 20, and 40 mg/L), Pb^2+^ (20, 60, 160, and 240 mg/L), and Cd^2+^ (0.5, 1, 2, and 4 mg/L) for 96 h. The cell density was measured every 24 h with 721-type ultraviolet spectrophotometer at the wavelength of 450 nm. Normal algae cells were set as the control group. The experiments were conducted in triplicate. The inhibition rate and median effective concentrations (EC_50_) were monitored by the following equation Equation 1, referring to Romero et al. (2020).


(1) $$\mathrm{Inhibitory}\;\mathrm{rate}\;\left(IR\right)=\left(1-\frac{Ntn-Nt0}{Ncn-Nc0}\right)\times 100\%$$

where N_t0_ represented the initial cell number of treatment group; N_tn_ was the cell number of the treatment group at different time; N_c0_ was the initial cell number of the control group; N_cn_ represented the cell number of the control group at different time.

### 2.3 Measurement of photosynthetic pigment contents

Photosynthetic pigments of *D. salina*, such as chlorophyll a, chlorophyll b, and carotenoids, were extracted according to acetone colorimetry (Xu et al., 2016; Cheng et al., 2021). In brief, after exposing to different concentrations of Cu^2+^ (5, 10, 20, and 40 mg/L), Pb^2+^ (20, 60, 160, and 240 mg/L), and Cd^2+^ (0.5, 1, 2, and 4 mg/L) for 72 h, 10 mL of algal solution in each group was centrifuged for 10 min at 4,000 rpm, and the supernatant was discarded. The algae pellet was extracted with 10 mL of 90% acetone at low temperature without light for 24 h and then centrifuged. The chlorophyll contents were measured at 663, 645, and 652 nm, respectively, by a spectrophotometer. Each test was performed in triplicate. The photosynthetic pigments were determined according to the following formula Equations 2–5, referring to Cheng et al. (2016) and Xu et al. (2016):


(2) $$\mathrm{Chla}\;\left(\mathrm{mg}/L\right)=12.7A663–2.69A645$$

(3) $$\mathrm{Chlb}\;\left(\mathrm{mg}/L\right)=22.9A_{645-}4.68A_{663}$$

(4) $$Ct\;\left(\mathrm{mg}/L\right)=\frac{A652\times 1000}{34.5}$$

(5) $$\mathrm{Car}\;\left(\mathrm{mg}/L\right)=A_{480}\times 4.0$$

where Chla, Chlb, Ct, and Car represented chlorophyll a, chlorophyll b, total chlorophyll, and carotenoid contents, respectively.

### 2.4 Determination of polysaccharides and protein

The anthrone-sulfuric acid colorimetry method was adopted to determine the content of intracellular soluble polysaccharides (Yu et al., 2019). After exposing to different concentrations of Cu^2+^ (5, 10, 20, and 40 mg/L), Pb^2+^ (20, 60, 160, and 240 mg/L), and Cd^2+^ (0.5, 1, 2, and 4 mg/L) for 72 h, 10 mL of *D. salina* samples were harvested by centrifuging at 2,000 rpm for 10 min, and then, algal pellet was washed with distilled water. The algal solution was repeatedly undergone freezing and thawing at −20°C three times and then was broken by ultrasound for 60 s with a model KQ5200DE CNC ultrasonic cleaner (Ultrasonic Instruments Corporation, Kunshan, JiangSu, China). The soluble polysaccharide extract was obtained by centrifuging at 4,000 rpm for 10 min, collecting the supernatant, and fixing with distilled water to 10 mL. Subsequently, 1 mL of the extract was added with 5 mL of anthrone reagent, boiled for 10 min, cooled in condensate water, and centrifuged. Finally, the supernatant was measured at a wavelength of 620 nm to determine the polysaccharide content.

In addition, the content of intracellular soluble protein was determined according to Coomassie brilliant blue method (Tang et al., 2016). After 72 h of exposure to different concentrations of heavy metals, the pellet was obtained by centrifuging from 10 mL samples of *D. salina* at 2,000 rpm for 20 min, suspended in phosphate buffer solution (PBS), disrupted by ultrasound for 60 s, and then centrifuged at 4,000 rpm for 20 min. Subsequently, 5 mL of Coomassie brilliant blue solution was added to 1 mL of the extract, mixed, and then stood for 2 min. Finally, the supernatant was measured at a wavelength of 595 nm, and the protein content was determined according to the bovine serum albumin (BSA) standard curve.

### 2.5 Determination of SOD, CAT activity, and MDA content

The activities of antioxidant enzymes including SOD and CAT and MDA content were performed using appropriate assay kits (Jiancheng Biological Corporation, Nanjing, Jiangsu, China). After exposure to different concentrations of the three heavy metals for 72 h, 10 mL of *D. salina* samples was centrifuged at 2,000 rpm for 10 min, the supernatant was discarded, and then, the cells were suspended with PBS (0.1 M, pH 7). Subsequently, the cells were vortex-oscillated for 3 min and centrifuged at 4,000 rpm for 10 min. Afterward, the supernatant was diluted with PBS to different concentrations, and then, the enzyme and substrate solution were added and mixed well and incubated at 37°C for 20 min. Finally, the enzyme solution was measured with microplate reader at 450 nm and 405 nm for SOD and CAT, respectively.

Moreover, MDA content was determined according to the 2-thiobarbituric acid (TBA) method (Chu et al., 2022; Amjadi et al., 2024). After exposure to different concentrations of the three heavy metals for 72 h, 10 mL of *D. salina* samples were centrifuged at 2,000 rpm for 10 min, and the supernatant was discarded; 0.1 mL of cells was added to 0.9 mL of 10% (w/v) thiobarbituric acid (TBA), then homogenized on ice with a homogenizer at 10,000 rpm for 10 s each time, 30 s interval three times. Then, the homogenate was centrifuged at 4,000 rpm for 10 min. Subsequently, 50 μL of the supernatant was added to 1 mL of working solution containing 20% TBA, followed by incubation at 95°C for 30 min for the color reaction. Afterward, the tubes underwent further centrifugation at 10,000 rpm for 10 min at 4°C, and the supernatant was measured at the absorbance of 532 nm, 600 nm, and 450 nm. The MDA content was calculated according to the following formula Equation 6, referring to Amjadi et al. (2024) and Chu et al. (2022).


(6) $$MDA\;\left(\mu \mathrm{mol}/L\right)=6.45\times \left(A_{532}-A_{600}\right)-0.56\times A_{450}$$

where A_450_, A_532_, and A_600_ are the absorbances at 450 nm, 532 nm, and 600 nm, respectively.

### 2.6 The removal optimization of heavy metals using box–Behnken design

Based on the response surface methodology (RSM), Box–Behnken design (BBD) was employed to identify the optimal parameters of *D. salina* for the removal rates of Cu^2+^, Pb^2+^, and Cd^2+^. According to BBD methodology, three variables including temperature (°C) (A), pH (B), and time (h) (C) were categorized into three levels (−1, 0, and 1). The temperature levels were set at 10°C, 30°C, and 50°C, pH values were 3, 5, and 7, and the time levels were 0, 6, and 12 h, as presented in Table 1. The contents of heavy metal ions were determined by atomic absorption spectrometry (Agilent, Germany). The removal efficiency (Y) was considered as the response. Design Expert 13.0.1 software was utilized to analyze the interactions of three variables using 3D surface plots and identify the design points located above these surfaces, indicating the optimum conditions for *D. salina* to remove heavy metals.

**Table 1:** Range of parameters for Cu^2+^, Pb^2+^, and Cd^2+^ removal by *Dunaliella salina.*

Variables units		Symbol		Coded Levels		
		Uncoded	Coded	-1	0	+1
Temperature	°C	X_1_	A	10	30	50
pH	-	X_2_	B	3	5	7
Time	h	X_3_	C	0	6	12

### 2.7 Metal ion distribution and adsorption mechanism

The concentrations of heavy metal ions were chemically analyzed after *D. salina* cells were exposed to different heavy metals for certain time, according to using spectrophotometer and referring to the method outlined by Elleuch et al. (2021a). In brief, 5 mL of microalgal culture was centrifuged at 6000 × g for 10 min to separate the supernatant and algal pellet. The supernatant was filtered through a 0.22 μm membrane filter. Then, 250 μL of concentrated HNO_3_ was added to the filtrate to determine the dissolved concentrations of Cu^2+^, Pb^2+^, and Cd^2+^ at an absorbance of 324.7 nm, 283.3 nm, and 228.8 nm, respectively, by atomic absorption spectroscopy (Thermo Scientific iCE™ 3,300). The removal rates of heavy metals by *D. salina* are calculated as follows Equation 7, referring to Elleuch, et al. (2021a):


(7) $$R\;\left(\%\right)=\left(C_{i}-C_{f}\right)/C_{i}\times 100\%$$

where R is the removal rate; C_i_ represents the initial concentration of metal ions; *C_f_* is the final concentration of metal ions after adsorption for certain time.

Additionally, the concentrations of intracellular or extracellular metals were determined. In brief, 5 mL of microalgal culture was centrifuged at 6000 × g for 10 min to separate the supernatant and algal pellet. The supernatant was filtered through a 0.22 μm membrane filter. Then, 250 μL of concentrated HNO_3_ was added to the filtrate to determine the dissolved concentrations of heavy metals. Afterward, the algal pellets were suspended in 2 mL of 0.02 M EDTA and shaken for 30 s to remove the adsorbed metals on the surface of algal cells. Then, the samples were centrifuged at 6000 × g for 10 min to further separate the supernatant and algae pellet. Subsequently, 250 μL of concentrated HNO_3_ was added to the supernatant, facilitating the quantification of extracellular concentrations of heavy metals. In addition, the precipitates underwent acid digestion with 250 μL of concentrated HNO_3_ in a water bath at 90°C for 1 h; the acid-treated sample was then diluted with Milli-Q water and used for the determination of intracellular concentration of heavy metals. The concentrations of heavy metals were measured at a wavelength of A_324.7_, A_283.3_, and A_228.8_ for Cu^2+^, Pb^2+^, and Cd^2+^, respectively, using an Atomic Absorption Spectroscopy (ICE 3000 series). Both extracellular and intracellular metal concentrations were expressed as ng/10^6^ cells.

### 2.8 Fourier transform infrared spectroscopy

Fourier transform infrared (FT-IR) spectroscopy was used to analyze the biosorption mechanism of the three heavy metals by *D. salina*. In brief, algal samples of *D. salina* were filtered through millipore filters; the algal sediments were rinsed with double-distilled water 2–3 times and then lyophilized for 24 h. Subsequently, algal samples were ground to a size of approximately 2 μm, and 1–2 mg of samples was mixed well with potassium bromide powder (100 mg, 200 mesh). Afterward, they were prepared transparent thin samples, and the functional groups on the cell surface of *D. salina* were determined within the range of 500–4,000 cm^−1^ using Fourier transform infrared spectroscopy (Nicolet, United States).

### 2.9 Statistical analysis

All the data were performed using one-way analysis of variance (ANOVA) followed by Tukey’s test in SPSS 22.0 software (SPSS, Inc., Chicago, IL). All experiments were carried out in triplicate, and data were expressed as mean ± SD. *p* < 0.05 was considered to be statistically significant.

## 3 Results

### 3.1 Effects of Cu^2+^, Pb^2+^, and Cd^2+^ on the growth of *Dunaliella salina*

**Figure 1:**
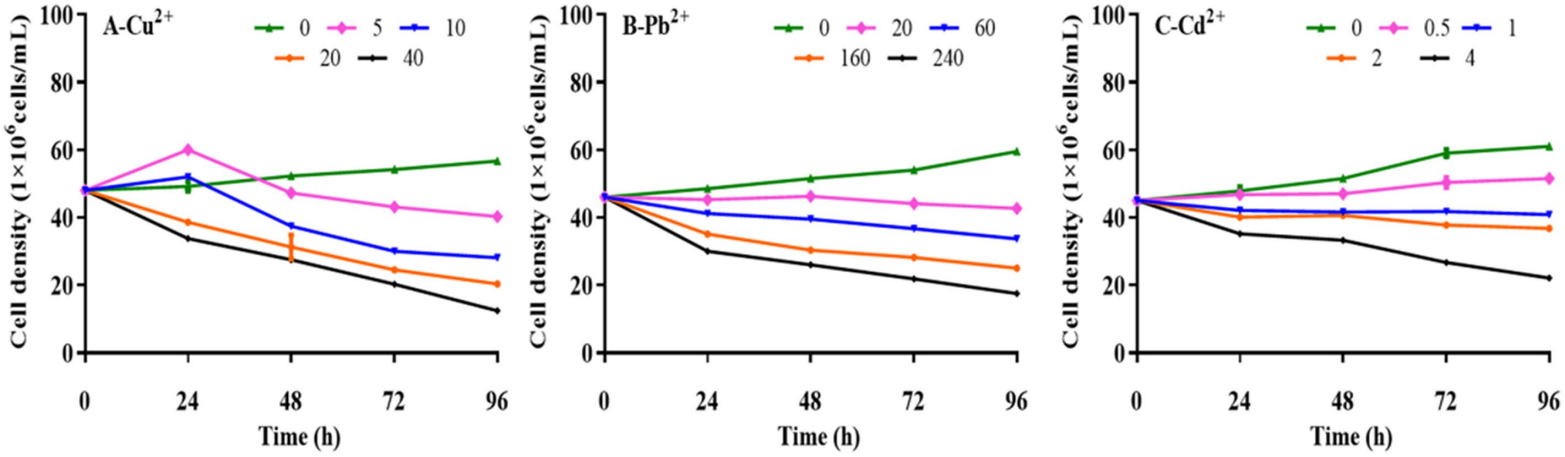
Effects of Cu^2+^, Pb^2+^, and Cd^2+^ on the growth of *Dunaliella salina.*

Kinetic analysis was performed to determine the effects of Cu^2+^, Pb^2+^, and Cd^2+^ on the growth of *D. salina.* As presented in Figure 1, the cell densities of *D. salina* increased from 4.92 × 10^7^ cells/ml to 5.45 × 10^7^ cells/ml and 5.20 × 10^7^ cells/ml at 5 mg/L and 20 mg/L of Cu^2+^ within 24 h, respectively, while decreased with the increasing time, demonstrating a hormesis effect (Calabrese and Agathokleous, 2023). However, high concentrations of Cu^2+^ (>10 mg/L) displayed remarkable inhibitory effects on cell proliferation with the increasing of exposure time, exhibiting a time- and concentration-dependent effect. The EC_50_ value of Cu^2+^ on algal cells at 72 h was 18.14 mg/L. Similarly, the cell densities decreased with increasing Pb and Cd concentration and exposure time, demonstrating obvious toxicity to *D. salina* in a concentration- and time-dependent manner. The EC_50_ values were calculated to be 160.37 mg/L and 3.32 mg/L for Pb and Cd at 72 h, respectively. The results indicated that cadmium was considered to be the most toxic substance of the three metals for *D. salina*.

### 3.2 Effects of Cu^2+^, Pb^2+^, and Cd^2+^ on photosynthetic pigments in *Dunaliella salina*

**Figure 2:**
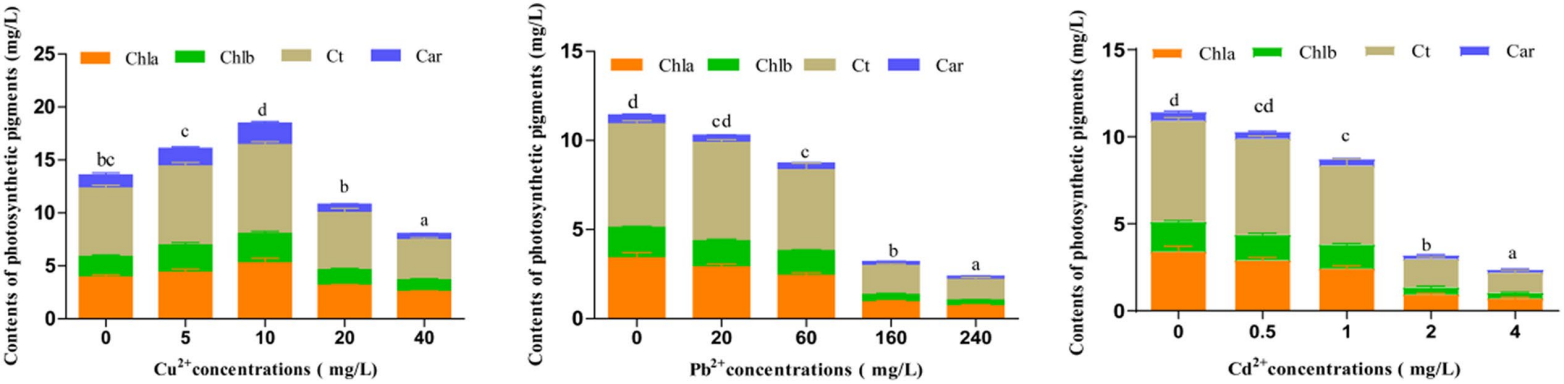
Effects of Cu^2+^, Pb^2+^, and Cd^2+^ on the photosynthetic pigments of *Dunaliella salina.* The different letters in each column represent the differences among different groups (*p* < 0.05).

Photosynthetic pigments are essential in assessing the photosynthetic capacity of plants or algae and the extent of environmental stress (Li et al., 2022). After exposing to different concentrations of Cu^2+^, Pb^2+^, and Cd^2+^ for 72 h, the contents of photosynthetic pigments, including chlorophyll a (Chla), chlorophyll b (Chlb), total chlorophyll (Ct), and carotenoids (Car), in *D. salina* were enhanced when Cu^2+^ concentration was under 10 mg/L, indicating that low dose of copper could trigger the accumulation of photosynthetic pigments, whereas these pigments declined with the increase in Cu^2+^ concentration (>10 mg/L), exhibiting a hormesis effect (Figure 2), which was consistent with the inhibitory effect. Conversely, the contents of photosynthetic pigments dramatically declined with increasing concentrations of Cd^2+^ and Pb^2+^ (*p* < 0.05), suggesting that cadmium and lead remarkably restrained the growth and synthesis of photosynthetic pigments in *D. salina.*

### 3.3 The contents of polysaccharide and protein

**Figure 3:**
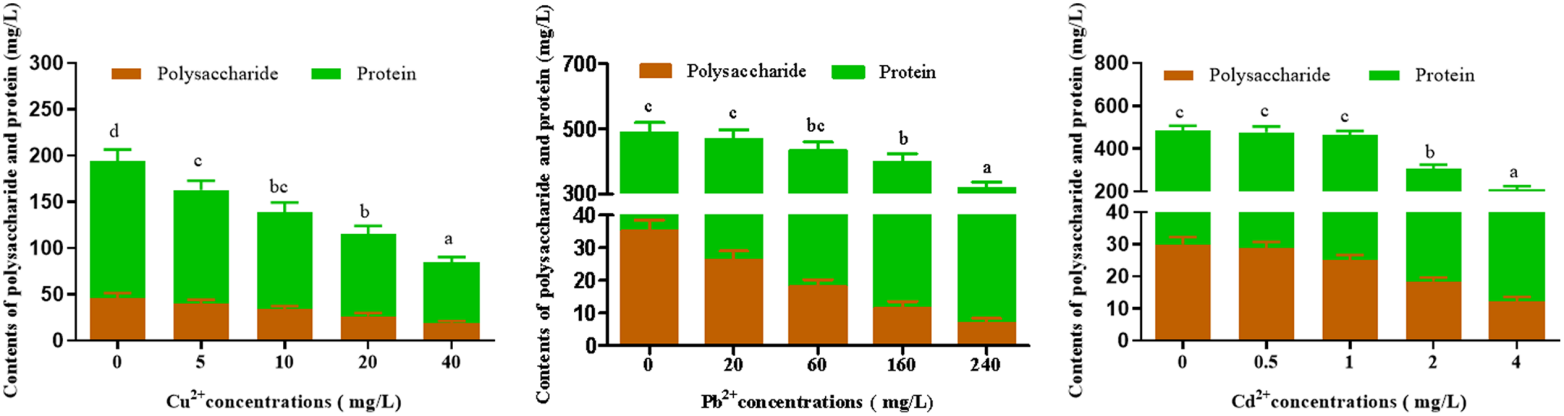
Effects of Cu^2+^, Pb^2+^, and Cd^2+^on the polysaccharides and protein contents of *Dunaliella salina.* The letters in each column represent significant difference among different groups (*p* < 0.05).

The influence of Cu^2+^, Pb^2+^, and Cd^2+^ stress on polysaccharide and protein contents of *D. salina* is presented in Figure 3. After 72 h of exposure, the obvious reduction in polysaccharide and protein contents was observed, which was correlated with the rise in three metal concentrations. Specifically, the contents of polysaccharide and protein decreased by 41.65 and 55%, 62.05 and 14.59%, 71.94 and 36.18% with the highest concentration of 40 mg/L Cu^2+^, 240 mg/L Pb^2+^, and 4 mg/L Cd^2+^, respectively.

### 3.4 The antioxidant response to heavy metals

**Figure 4:**
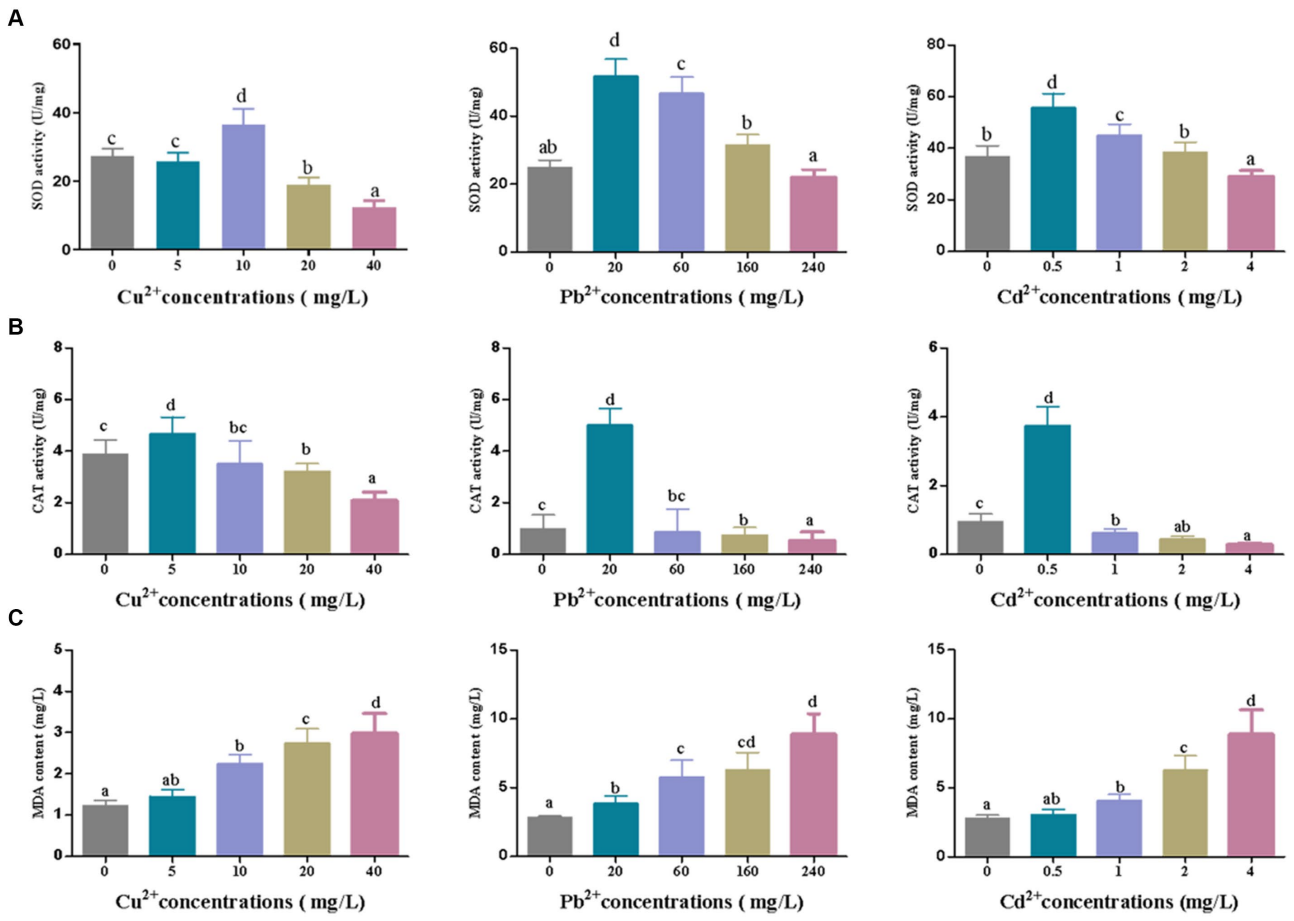
The changes of **(A)** SOD and **(B)** CAT activities and **(C)** MDA content in *Dunaliella salina* under different concentrations of Cu^2+^, Pb^2+^, and Cd^2+^ stress. The different letters in each column represent significant difference among different groups (*p* < 0.05).

Under the adverse environment, algae produce a large number of ROS in cells, which can be decomposed by the antioxidant enzymes such as SOD, CAT, GPX, and APX via eliminating O2 and H_2_O_2_, to prevent cells against oxidative damage (Liu et al., 2018; Sun et al., 2022). Moreover, oxidative damage can cause lipid peroxidation and produce malondialdehyde (MDA), a biomarker of oxidative damage (Lu L. et al., 2015). As shown in Figure 4, the activities of SOD and CAT in *D. salina* increased sharply and then declined as the concentrations of Cu^2+^, Pb^2+^, and Cd^2+^ increased, with the highest activities of SOD and CAT appearing at 10 mg/L of Cu^2+^, 20 mg/L of Pb^2+^, and 0.5 mg/L of Cd^2+^, respectively, after 72 h of exposure. Simultaneously, MDA content increased continuously under the metal stress, reaching the peak at the highest dose of metal ions (40 mg/L of Cu^2+^, 240 mg/L of Pb^2+^, and 4 mg/L of Cd^2+^). In short, enhancement of SOD and CAT activities to scavenge ROS can be regarded as a rapid response to heavy metal stress. Then, the activities of SOD and CAT declined, and the MDA level increased with the increasing concentrations of heavy metals, revealing that heavy metals caused oxidative damage to algal cells.

### 3.5 The correlationship analysis and PCA plot

**Figure 5:**
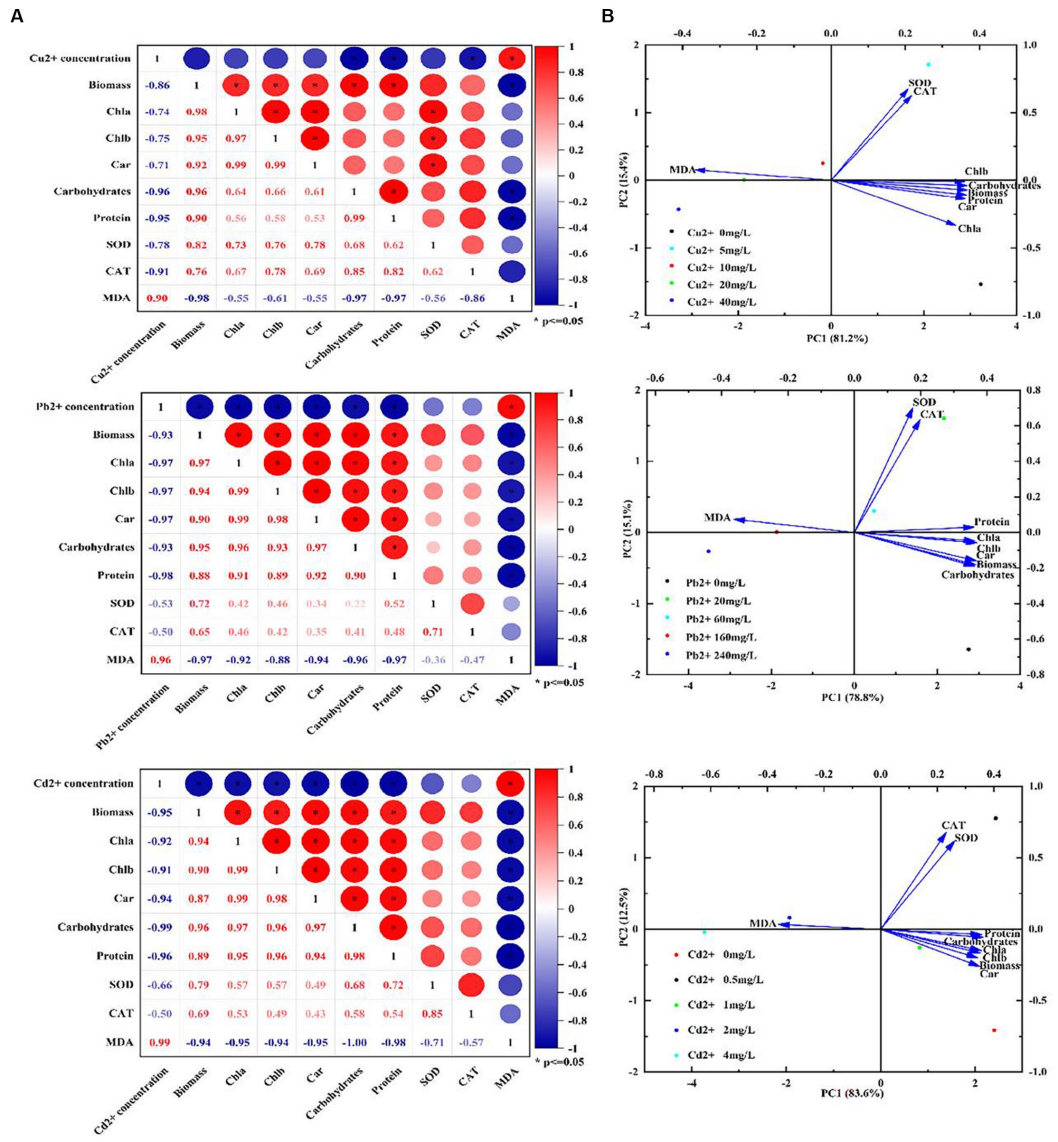
Correlation analysis and PCA biplot of different parameters under Cu^2+^, Pb^2+^, and Cd^2+^ stress. **(A)** Correlation analysis. **(B)** PCA biplot.

Additionally, the correlationship analysis and principal component analysis (PCA) were also performed to explore the relationship between heavy metal concentrations and physiological characteristics or antioxidant system under copper, lead, and cadmium stress. As shown in Figure 5A, there was a significant negative correlation between heavy metal concentrations and biochemical parameters (e.g., biomass (cell density), Chl a, Chl b, Car, carbohydrates, and protein) or antioxidant system (SOD and CAT) with the correlation coefficients ranged from −0.50 to −0.99, whereas a positive correlation existed between the ion concentrations and MDA content. In addition, the biochemical parameters were found to be positively correlated with SOD and CAT activities, with the correlation coefficient ranges of 0.62–0.85, 0.22–0.52, and 0.43–0.72 after exposing to copper, lead, and cadmium, respectively, indicating that *D. salina* was more sensitive to copper stress through exerting more significant correlation with antioxidant response under copper stress. Moreover, the intercorrelation between these parameters was also explored using PCA analysis. As shown in Figure 5B, the statistical data in PC1 and PC2 accounted for 81.2 and 15.4%, 78.8 and 15.1%, 83.6 and 12.5% of the variances in Cu-, Pb-, and Cd-treated algal cells, respectively. These parameters occupied different positions and presented diverse relationship with each other. It was worth noting that all parameters were situated at the positive side of PC1, except for MDA. More fascinatingly, there was a remarkable covariance among Chla, Chlb, Car, carbohydrates, protein, and SOD and CAT. Furthermore, the parameters of all the biochemical parameters were located on the positive side of PC1 and negative side of PC2, while SOD and CAT were the most correlated and oriented on both positive sides of the biplot in Cu^2+^-treated cells. Therefore, it was proposed that the photosynthetic pigments, intracellular carbohydrates, and proteins in algal cells were the crucial substances for physiological response under heavy metal stress, whose changes may be considered as biomarkers for cytotoxicity assessment (Kovaleski et al., 2022).

### 3.6 The removal optimization of *Dunaliella salina* on heavy metals

Equations 8–10 were obtained based on a a second-order polynomial model (Shao et al., 2011). The removal efficiency (Y) of Cu^2+^, Pb^2+^, and Cd^2+^ was regressed by a second-order polynomial model, according to the following equations:


(8) $$\begin{array}{l} Y\;\left({Cu}^{2+}\right)=89.70+1.27A+4.71B+0.9056C+0.30\mathrm{AB}-1.23\mathrm{AC}\\ \;-0.2722BC-15.35A^{2}-19.42B^{2}-11.62C^{2} \end{array}$$

(9) $$\begin{array}{l} Y\;\left({Pb}^{2+}\right)=87.20+2.19A-8.55B-1.36C-3.16\mathrm{AB}\\ \;-2.12BC-9.41A^{2}-22.09B^{2}-7.84C^{2} \end{array}$$

(10) $$\begin{array}{l} Y\;\left({Cd}^{2+}\right)=72.90+2.07A+6.53B+1.18C-0.2889\mathrm{AB}+1.35\mathrm{AC}\\ \;+0.2889BC-12.45A^{2}-11.18B^{2}-7.25C^{2} \end{array}$$

The statistical significance was determined by ANOVA analysis with Design Expert 13.0.1 software. The *p*-values for the quadratic terms of each factor implied a high dependence on the tested factors. The surface plot analysis revealed remarkable interactions between each pair of variables, elucidating the ANOVA outcomes (Table 2).

**Table 2:** ANOVA analysis for the regression model of Cu^2+^, Pb^2+^, and Cd^2+^ removal.

Metal	Source	Sum of squares	Df.	Mean square	*F*-value	*P*-value	Significant
Cu^2+^	Model	3286.93	9	365.21	474.17	<0.0001	*
	A	11.70	1	11.70	15.20	0.0059	*
	B	177.66	1	177.66	230.66	<0.0001	*
	C	5.90	1	5.90	7.67	0.0277	*
	AB	0.4050	1	0.4050	0.5258	0.4919	
	AC	6.00	1	6.00	7.79	0.0268	*
	BC	0.3335	1	0.3335	0.4330	0.5316	
	A2	992.09	1	992.09	1288.07	<0.0001	*
	B2	789.76	1	789.76	1025.37	<0.0001	*
	C2	569.01	1	569.01	738.77	<0.0001	*
	Residual	5.39	7	0.7702			
	Lack of fit	5.39	3	1.80			
	Pure error	0.0000	4	0.0000			
	Cor total	3292.32	16				
Pb^2+^	Model	3610.93	9	401.21	25.26	0.0002	*
	A	38.28	1	38.28	2.14	0.1645	
	B	584.82	1	584.82	38.82	0.0005	*
	C	14.85	1	14.85	0.9352	0.3657	
	AB	39.82	1	39.82	2.51	0.1573	
	AC	0.0000	1	0.0000	0.0000	1.0000	
	BC	18.06	1	18.06	1.14	0.3216	
	A2	372.83	1	372.83	23.48	0.0019	*
	B2	2053.67	1	2053.67	129.32	<0.0001	*
	C2	258.80	1	258.80	16.30	0.0050	*
	Residual	111.17	7	15.88			
	Lack of fit	111.17	3	37.06			
	Pure error	0.0000	4	0.0000			
	Cor total	3722.10	16				
Cd^2+^	Model	1997.55	9	221.95	258.34	<0.0001	*
	A	30.92	1	30.92	35.99	0.0005	*
	B	340.61	1	340.61	396.45	<0.0001	*
	C	9.99	1	9.99	11.63	0.0113	*
	AB	0.3756	1	0.3756	0.4371	0.5397	
	AC	7.29	1	7.29	8.49	0.0226	*
	BC	0.3756	1	0.3756	0.4371	0.5297	
	A2	652.64	1	652.64	759.66	<0.0001	*
	B2	261.77	1	261.77	304.69	<0.0001	*
	C2	221.32	1	221.32	257.61	<0.0001	*
	Residual	6.01	7	0.8591			
	Lack of fit	6.01	3	2.00			
	Pure error	0.0000	4	0.0000			
	Cor total	2003.56	16				

*Statistically significant at 95% of confidence level.

**Figure 6:**
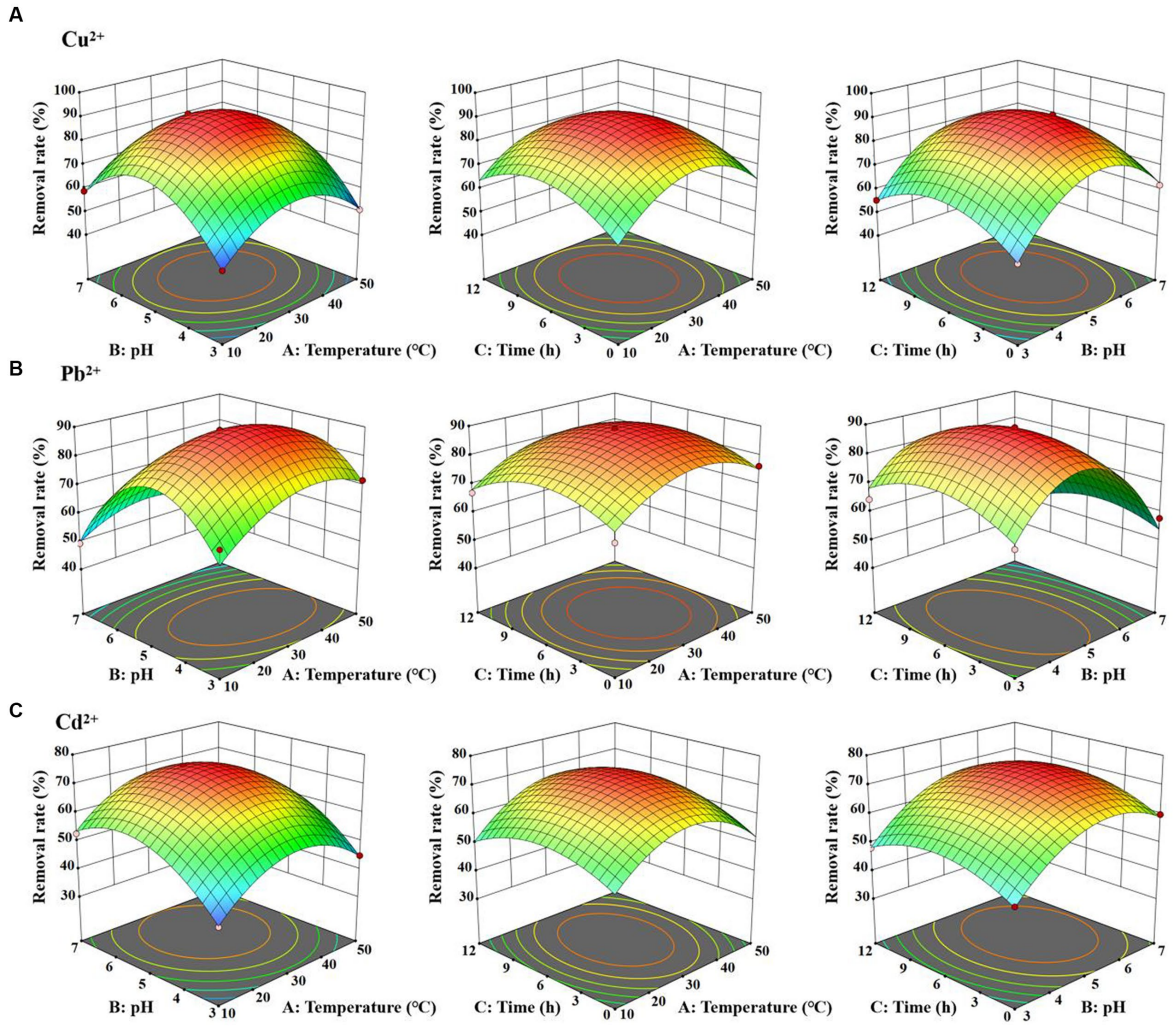
3D plot for the interactive effect of temperature, pH and exposure time for the removal efficiency of Cu^2+^ **(A)**, Pb^2+^ **(B)**, and Cd^2+^ **(C)** by *Dunaliella salina.*

Moreover, the interrelations between the removal efficiency of copper, lead, and cadmium and the levels of each experimental variable were graphically portrayed with 3D response surface plots. As shown in Figure 6, with the rise in temperature from 10°C to 30°C, pH from 3 to 5, and exposure time from 0 h to 6 h, the removal rates of Cu^2+^, Pb^2+^, and Cd^2+^ significantly increased. Specifically, the highest removal rate of Cu^2+^ achieved 89.70% at temperature of 30°C, pH of 5, and an adsorption duration of 6 h. Simultaneously, the optimum removal efficiency of Pb^2+^ and Cd^2+^ achieved 87.20 and 72.90%, respectively, with the same temperature of 30°C, pH of 6, and the duration of exposure of 6 h. The p-values for all three models were less than 0.0005, confirming a statistical significance. In short, BBD reliably simulated the removal of Cu^2+^, Pb^2+^, and Cd^2+^ by *D. salina*, optimizing their effective factors.

### 3.7 Metal ion distribution and removal mechanism

**Figure 7:**
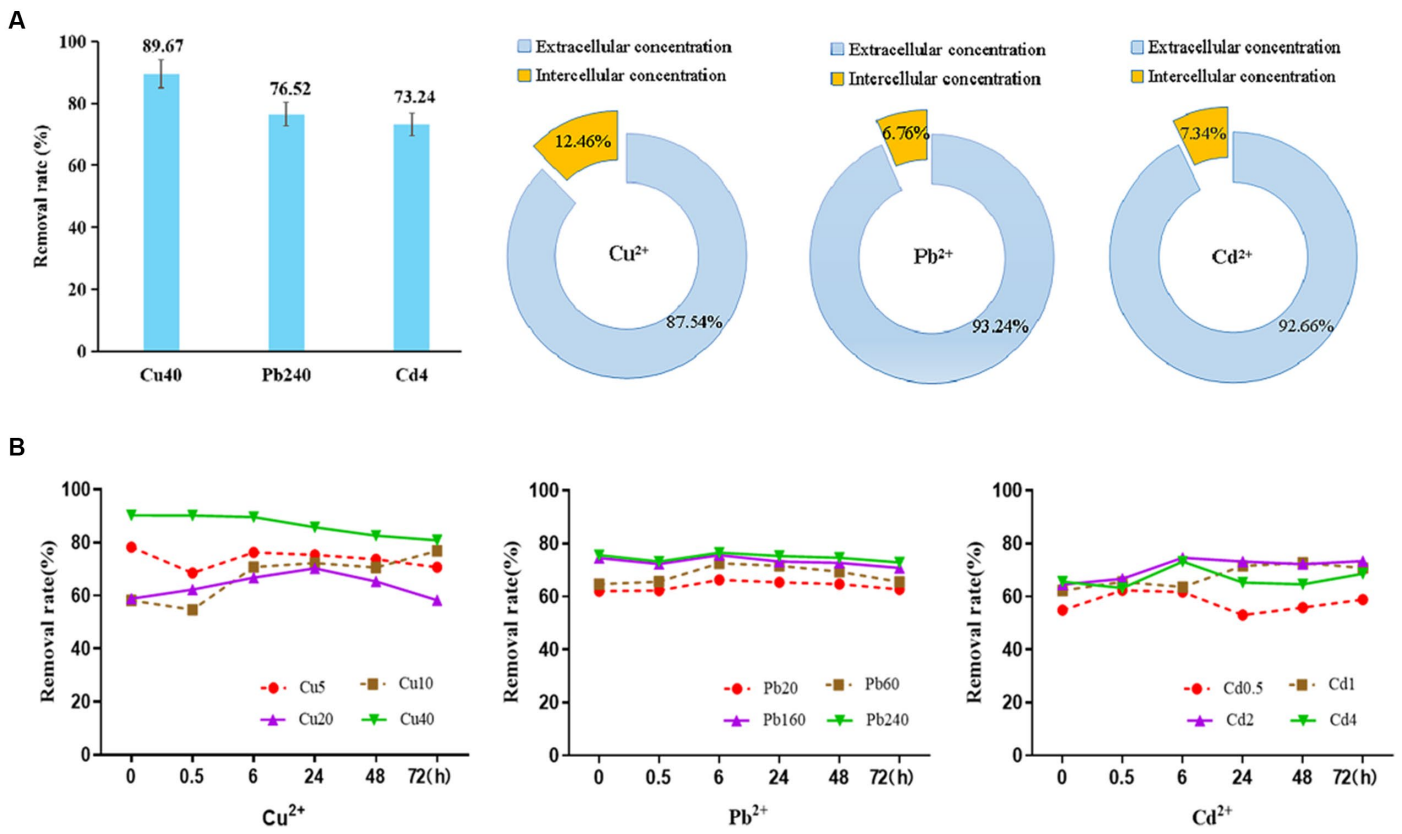
The adsorption curve of Cu^2+^, Pb^2+^, and Cd^2+^ by *Dunaliella salina* under different time and concentration and their extracellular or intracellular concentration.

Based on the results of BBD analysis, the capacities of *D. salina* to remove Cu, Pb, and Cd ions were evaluated. After exposure to different heavy metals with the highest dose of 40 mg/L Cu^2+^, 240 mg/L Pb^2+^, and 4 mg/L Cd^2+^ for 6 h, the concentrations of dissolved metal ions in the culture were considered as the relative removal amount of metal ions. Specifically, the removal rates of Cu, Pb, and Cd ions by *D. salina* were 89.67, 76.52, and 73.24%, respectively. It is noteworthy that *D. salina* exhibited higher removal capacity for Cu ions. Moreover, the extracellular and intracellular uptake of the metal ions were further determined. The extracellular concentrations of Cu^2+^, Pb^2+^, and Cd^2+^ accounted for a striking proportion of 87.54, 93.24, and 92.66%, respectively, indicating a surface bioadsorption mechanism. Meanwhile, the intracellular contents of Cu^2+^, Pb^2+^, and Cd^2+^ occupied 12.46, 6.76, and 7.34%, respectively, demonstrating a bioaccumulation mechanism (Figure 7A). In this study, both surface bioadsorption and intracellular bioaccumulation were involved in the uptake of copper, lead, and cadmium ions by *D. salina,* which was consistent with zinc adsorption by *Dunaliella* sp. AL-1, *Chlorella pyrenoidosa,* and *Scenedesmus obliquus* (Novák et al., 2020; Elleuch et al., 2021a). Elleuch et al. (2021b) reported Pb removal capacities of 50–95% using *D. salina,* with surface biosorption of 50.66% and intracellular bioaccumulation of 49.34%.

To further elaborate the bioadsorption mechanism, the initial concentrations of heavy metals and action time were evaluated on the removal rate and the adsorption process. As shown in Figure 7B, the removal of Cu^2+^ achieved the highest of 78.9% instantaneously at the dose of 5 mg/L at the beginning and then decreased within 30 min dramatically, followed by a steady rise within 24 h. It can be inferred that the surface biosorption may be the main removal mechanism at first. After the metal binding sites on the surface of cells were captured by the metal ions, the excessive ions may be bioaccumulated into the cells, leading to a relatively high removal rate. Meanwhile, the removal efficiencies of Cu^2+^ at 40 mg/L were obviously higher than other concentrations, with the range of 80.9–90.3%. At this time, both surface biosorption and intracellular accumulation were all involved in the removal of high level of copper ions. On the contrary, the removal rates of Pb^2+^ and Cd^2+^ were relatively stable with the increasing concentrations and time. The adsorption of Pb^2+^ by *D. salina* is a rapid process. Within 6 h, the maximum removal rate of Pb^2+^ was 76.5% and then leveled off steadily. Meanwhile, the bioadsorption of Cd^2+^ was a slow rise process which is performed for 6 h; the removal rates were from 61.7 to 74.6% at the concentration range of 0.5–4 mg/L. Subsequently, the adsorption rate decreased and then rose slowly. Taken together, it is speculated that surface bioadsorption and intracellular bioaccumulation are both involved in the removal mechanism of Cu^2+^, Pb^2+^, and Cd^2+^ by *D. salina.* To be specific, the surface adsorption may mediate the removal of heavy metals before 6 h, whereas the intracellular bioaccumulation may be the main removal mechanism within 6–72 h.

### 3.8 The infrared absorption spectrum of Cu^2+^, Pb^2+^, and Cd^2+^ by *Dunaliella salina*

**Figure 8:**
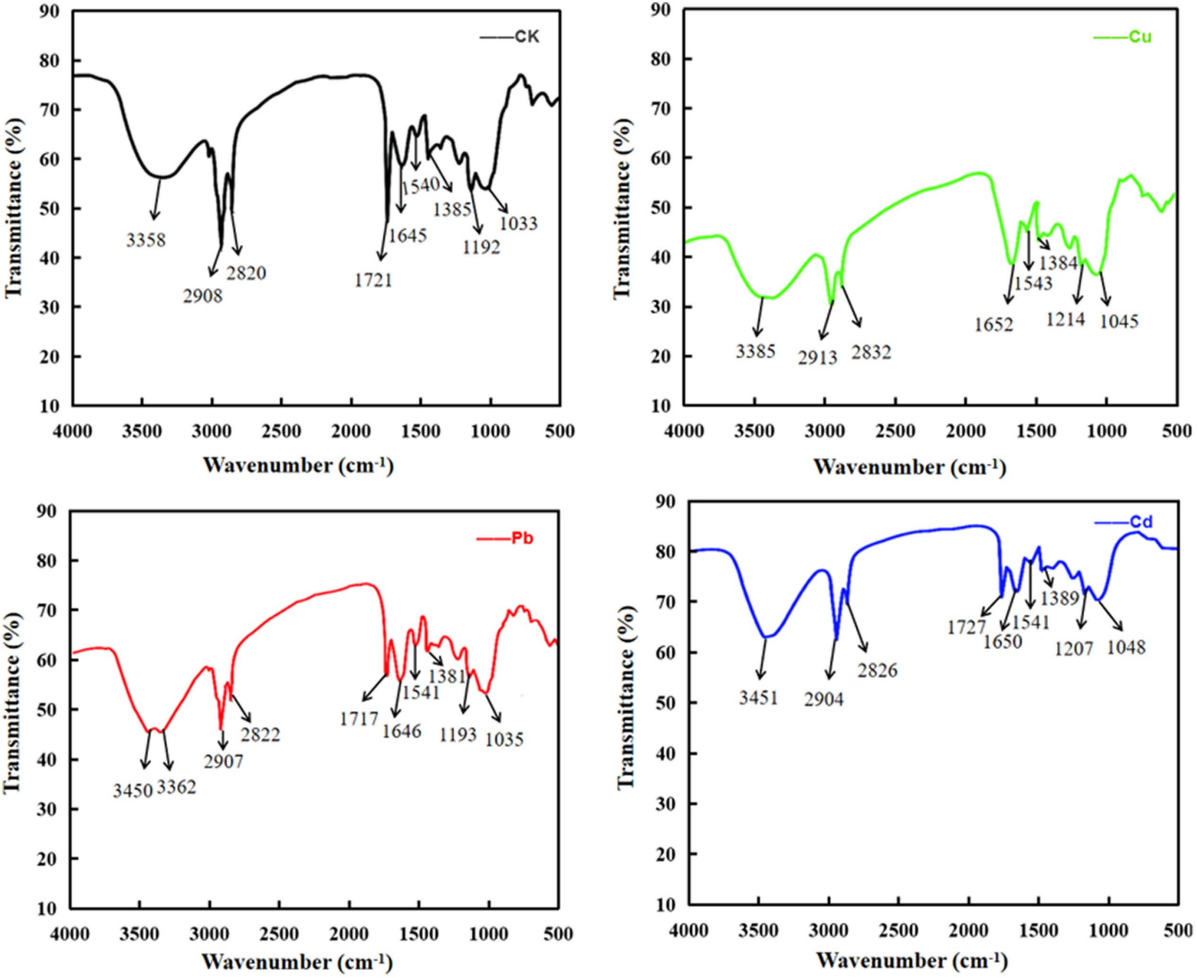
The infrared absorption spectrum of Cu, Pb, and Cd ions by *Dunaliella salina*.

It is noteworthy that polysaccharides, proteins, lipids, and nucleic acids are composed of multiple functional groups on the surface of plants or algae, especially hydroxyl, carboxylate, phosphate, and amino group for metal ion adsorption via binding to cations, and the mechanism of adsorption may be mediated by ion-exchange, complexation, chelation, and coordination (Shen et al., 2018; Jiang et al., 2023). To detect the differences that different metal ions interact with functional groups of cellular membrane, FTIR spectroscopy was used to analyze the absorption mechanism of *D. salina* before and after copper, lead, and cadmium exposure. As shown in Table 3, obvious changes were discovered in the characteristic absorption peaks of hydroxyl, carboxylic acid, amine, and amino groups, belonging to lipids, proteins, and carbohydrates. Specifically, the absorption peaks at 3385 cm^−1^ and 3,451 cm^−1^ were due to the stretching vibration of hydroxyl (-OH) and amidogen group (-NH) after exposing to copper and cadmium, respectively, whereas double peaks attributed to stretching vibrations at 3450 cm^−1^ and 3,362 cm^−1^ appeared in lead-treated algal cells, which were assigned to -OH and amino group (-NH_2_). All the peaks in the range of 3,639–3,029 cm^−1^ exhibited the presence of polysaccharides, proteins, and water. The changes in adsorption peaks discovered in the range of 3,012–2,809 cm^−1^ displayed the stretching vibrations of methyl group (-CH_3_) and methylene group (-CH_2_). Peaks discovered at 2880 and 2,650 cm^−1^ may be attributed to the aldehyde group (-CHO) of lipids. Meanwhile, the peaks at 1655–1638 cm^−1^ and 1,545–1,540 cm^−1^ may be due to the stretching vibrations of carboxyl (-C=O) and amine groups (−R2-NH) of proteins in favor of interacting with metal ions. The peaks at 1425–1330 cm^−1^ and 1,356–1,191 cm^−1^ may be attributed to the carboxyl (-COOH), phosphorous (-P=O), and sulfur (-S=O) groups of lipids. The peaks with the range of 1,072–980 cm^−1^ were attributed to the stretching vibration of C-C and C-O groups, which were abundant in polysaccharides (Figure 8). These functional groups may be involved in the interaction of membrane polysaccharides and lipids with heavy metal ions. Similarly, Elleuch et al. (2021b) found that the intracellular bioaccumulation and surface adsorption of Zn, Pb, and Cr ions were contributed to the functional groups on the cell wall, including hydroxyl, carboxyl, amino, phosphate, and sulfhydryl groups.

**Table 3:** Summary of wave numbers and corresponding functional groups.

Wavenumber (cm^−1^)				Wavenumber range (cm^−1^)	Functional groups	Substance
Untreated control	After Cu^2+^ exposure	After Pb^2+^ exposure	After Cd^2+^ exposure			
3,358	3,385	3450/3362	3,451	3,639–3,029	υ O-H/υ N-H	Water, Protein, Polysaccharides
2,908	2,913	2,907	2,904	3,012–2,809	υ_as_ CH2, CH3	Lipids
2,820	2,832	2,822	2,826	2,880–2,650	υ CHO	Lipids
1721	–	1717	1727	1725–1705	υ C=O	Lipids
1,645	1,652	1,646	1,650	1,655–1,638/ 1,680–1,600	υ C=O	Protein (amide I)
1,540	1,543	1,541	1,541	1,545–1,540	δ N-H, υ C-N	Protein (amide II)
1,385	1,384	1,381	1,389	1,425–1,330	υ COOH	Lipids
1,192	1,214	1,193	1,207	1,356–1,191	υ_as_ P=O, S=O	Lipids
1,033	1,045	1,035	1,048	1,072–980	υ C-O/υ Si-O	Polysaccharides

^a^Band assignments based on Liu et al. (2018) and EI-Naggar et al. (2018). υ, symmetric stretch; υ_as_, asymmetric stretch; δ = symmetric deformation (bend).

## 4 Discussion

To the best of our knowledge, most of the metals (e.g., Cu^2+^, Zn^2+^, Mg^2+^, and Fe^2+^) are not only indispensable trace elements for plants or algae but also crucial cofactors for chlorophyll and multitude of enzymes. Their pivotal role in diverse physiological and biochemical processes cannot be understated, encompassing photosynthesis, respiration, signal transduction, and the antioxidant system, laying a solid foundation for plant growth and metabolisms (Moenne et al., 2016; Maleki et al., 2017). An increasing number of reports have indicated that heavy metals can restrain the growth of various algae and influence their physiological function, accompanied by the changes in photosynthetic pigments, polysaccharides, and protein (Chen et al., 2012; Bosnir et al., 2013; Cao et al., 2015b; Cheng et al., 2016; Mu et al., 2018; Bhattacharya, 2020; Nowicka et al., 2020; Wang et al., 2021; Mo et al., 2022). However, El Agawany et al. (2021) indicated that protein content of *Dunaliella tertiolecta* increased under low concentrations of Ni^+^, Zn^2+^, and Cu^2+^, whereas high concentrations of heavy metals impeded protein synthesis. In this study, low concentration of Cu ions (<10 mg/L) can accelerate the growth of *D. salina* and stimulate the accumulation of photosynthetic pigments, exhibiting a significant hormesis effect. However, high concentrations of heavy metals (Cu, Pb, and Cd ions) significantly impeded the growth of algae and suppressed the synthesis of photosynthetic pigments, polysaccharide, and protein, with a significant negative correlation between metal concentrations and the biochemical parameters, which is consistent with the findings reported by Amjadi et al. (2024). Furthermore, Ling et al. (2021) discovered a large amount of differentially expressed genes (DEGs)-encoding proteins relevant to photosynthesis, carbon assimilation, and carbohydrate metabolism were significantly upregulated in the Cu-treated group, including encoding PSI protein complex (PsaC and PsaE), PSII protein complex (PsbA, PsbB, PsbC, PsbD, PsbE, PsbH, PsbM, PsbP, PsbO, PsbQ, and PsbZ), chlorophyll a/b binding protein (LHCA3 and LHCB4), and cytochrome b6/f complex (PetB and PetC). In addition, RbcL gene-encoding Rubisco, participating in carbon assimilation, fixing, and photosynthesis, was also upregulated. It was claimed that heavy metals, at high concentrations, could potentially replace magnesium ions, a crucial component of chlorophyll core structure. This replacement not only affects the chlorophyll content but also is intricately linked to a reduction in the uptake of magnesium and nitrogen, which influences the photosynthesis and physiological metabolism (Srivastava et al., 2021).

Nevertheless, the accumulation of heavy metals universally intensifies lipid peroxidation and stimulates the generation of reactive oxygen species (ROS), ultimately leading to membrane degradation and oxidative stress, further disrupting the biochemical and metabolic activities of cells (Alho et al., 2019; Chu et al., 2022). Correspondingly, the detoxification of aquatic plants against ROS in response to heavy metal stress is carried out through antioxidant defense system, such as superoxide dismutases (SOD), catalases (CAT), glutathione peroxidases (GPX), peroxidase (POD), glutathione S-transferase (GST), glutathione reductase (GR), ascorbate peroxidase (APX), and glutathione (GSH), which can prevent the generation of free radicals and alleviate the oxidative damage to microalgae (Dao et al., 2017; Marella et al., 2020; Zhang et al., 2021; Zeng et al., 2022). It was indicated that high concentrations of Cu^2+^ and Cd^2+^ exhibited inhibitory effect on *Chlorella vulgaris* and triggered the release of ROS, leading to a severe oxidative stress with the decrease in CAT and SOD activities (Chen et al., 2016; Movafeghi et al., 2019). Similarly, Cao et al. (2015a,b) discovered that with increasing concentration of Cu and Pb, POD activity and MDA content gradually elevated in *Cladophora,* whereas SOD activity reduced and CAT activity exhibited no significant change. Further study indicated that the accumulation of Cu and Pb in *Cladophora* was strongly associated with the contents of total soluble sugar, Chla+Chlb, the activity of POD, the levels of MDA, and Metallothionein (MT), while the level of Zn was strictly relevant to the relative growth rate (RGR) and MDA content in *Cladophora*. POD and MT may be of great importance in the survival of *Cladophora* under Pb stress. These phenomena also occurred in our experiments. High concentrations of Cu, Pb, and Cd may lead to oxidative damage to *D. salina* with MDA content which is continuously increased. Reversely, SOD and CAT activities rose first and then decreased, indicating that these antioxidant enzymes seemed to be biomarkers for assessing the oxidative stress to plants or algae under diverse stress. The concentrations of Cu, Pb, and Cd ions were found to be negatively correlated with the activities of SOD and CAT, while a positive correlationship existed with MDA content. Moreover, our previous study revealed that low copper stress could trigger the expressions of the unigenes, encoding Cu/Zn-SOD and CAT and heat shock proteins in *D. salina* to defend against oxidative damage (Ling et al., 2021).

It is worth highlighting that algae exhibit a promising technique for bioremediation, facilitating the removal of heavy metals from wastewater. Compared with other bioremediation methods, algal bioremediation has several advantages, such as capable of handling wastewater with higher metal concentrations and regenerating and recycling biomass in multiple adsorption/desorption cycles. It is noteworthy that they exhibit high uptake capacity in removing heavy metals, making it both effective and cost-efficient (Romera et al., 2006; Brinza et al., 2007; Ajayan et al., 2011; Poo et al., 2018). Khan et al. (2022, 2023) discovered that *Vaucheria debaryana* and *Cladophora glomerata* had superior removal efficiency on heavy metals, such as Pb, Cd, Ni, and Cr. In addition, it cannot be ignored that the biosorption and removal efficiency of algae on heavy metal ions may be relevant to multiple factors, such as pH, temperature, algal biomass, and ionic concentration. As one of the most commonly used response surface methods (RSM), Box–Behnken design (BBD) is ideal for seeking the optimal conditions of adsorption process (Moon et al., 2013; Mutalik et al., 2021; Pascual et al., 2022; Ramesh et al., 2023; Zhao et al., 2023). For instance, Zeng et al. (2022) reported that the peak adsorption of *Microcystis aeruginosa* powder on Cu^2+^, Cd^2+^, and Ni^+^ was 83.24, 92.00, and 88.67% under the initial concentration of Cu^2+^, Cd^2+^, and Ni^+^ of 25 mg/L, 5 mg/L,and 15 mg/L, the temperature of 30°C, 35°C, pH value of 8, 8, and 7, and the adsorption time of 5 h, 4 h, and 1 h, respectively. Amjadi et al. (2024) claimed that the maximum adsorption of lead was 98.69% by *Haematococcus pluvialis* under the optimal conditions of adsorbent dose of 1 g/L, lead concentration of 25 mg/L, pH of 6, temperature of 25°C, and exposure time of 120 min. Among these conditions, pH is one of the most important environmental factors that affect the adsorption and removal of heavy metals by algae, especially influencing the solubility of heavy metal ions and the metal binding activity on the surface of algal cells. Generally, acidic conditions (pH 3.0–6.0) are more conducive to bioadsorption of heavy metal ions by algae (Ramesh et al., 2023). It is known that *D. salina* is a type of halophilic and eurythermic organism, with a strong adaptability to the growing environment. *D. salina* can survive and reproduce in a wide range of pH 5.5–11.5 and temperature of 4–40°C. Zhang et al. (2022a) claimed that *D. salina* obtained higher biomass and Fv/Fm values at pH 5.5 and 7.5 of culture medium than those at pH 9.5 and 11.5 due to the enhancement of photosynthesis. In this study, *D. salina* cells were domesticated at pH 3–7, temperature of 10–50°C, and duration of 0–12 h to optimize the adsorption conditions based on BBD analysis. The maximum removal efficiencies of Cu, Pb, and Cd ions achieved 89.70, 87.20, and 72.9%, respectively, under the optimal adsorption conditions, including temperature of 30°C, pH of 5–6, and an adsorption duration of 6 h. Jayakumar et al. (2022) obtained the optimal conditions of Cd^2+^ and Zn^2+^ removal by *Sargassum polycystum* according to BBD, with pH 4.65 and pH 5.7, biosorbent mass of 1.8 g/L and 1.2 g/L, and shaking speed of 76 rpm and 125 rpm, respectively. Notably, the optimal temperature range of bioremediation is narrow due to the fact that the biosorption process is endothermic and the removal efficiency enhanced along with the increase in temperature. However, high temperature may destroy cell structure and some active sites, lowering the removal rate (Zhao et al., 2023). Therefore, in the practical settings, *D. salina* cells can be domesticated to grow at pH of 5–6 and temperature of 25–30°C to adsorp the heavy metals from the wastewater, achieving a higher removal efficiency of heavy metals.

Further biosorption mechanism indicated that both surface bioadsorption and intracellular bioaccumulation are involved in the removal of Cu, Pb, and Cd ions. To be specific, the removal of Cu ions increased instantaneously at low concentration and then decreased within 30 min dramatically. This may be due to the reason that the initial adsorption of copper is a rapid process, with a certain amount of Cu ions in the algal culture immediately adsorbed on the cell surface of *D. salina* through electrostatic adsorption, ion exchange, and complexation, leading to a significant increase in the removal of Cu (Blaby-Haas and Merchant, 2012). Nevertheless, with the cell surface occupied by the metal ions, the residual Cu ions in the medium are relatively higher, which is equivalent to the decrease in removal efficiency. Subsequently, with the increasing concentration of Cu ions, the removal rates of Cu increased steadily because copper ions adsorbed on the cell surface of *D. salina* were transported, bioaccumulated intracellularly, and mediated by copper transporters or chaperones to participate in a series of physiological activities, promoting the growth and photosynthesis, especially at the concentrations of 5 mg/L and 10 mg/L of Cu^2+^. As a result, the removal rates increased slowly over time (Monteiro et al., 2012). After achieving a dynamic equilibrium, the adsorption rate dropped off gradually (Al-Homaidan et al., 2014). Meanwhile, the removal efficiencies of Cu^2+^ at 40 mg/L were obviously higher than other concentrations, with a range of 80.9–90.3%. On one hand, a large amount of copper ions can be transported and accumulated through surface adsorption and intracellular bioaccumulation, leading to an increase in the removal rate of Cu ions. On the other hand, higher dose of copper may destroy the cell structure of *D. salina*, causing the breakage of cells and blasting into pieces. Consequently, more metal binding sites on the surface were disclosed and adsorbed a great amount of copper ions, thereby enhancing the removal efficiency (Znad et al., 2022). On the contrary, the removal rates of Pb^2+^ and Cd^2+^ were relatively stable with the increasing concentrations and time. The adsorption of Pb^2+^ by *D. salina* is a rapid process. Within 6 h, the maximum removal rate of Pb^2+^ was 76.5% and then decreased steadily. This phenomenon may be attributed to the predominant surface bioadsorption at first and then attributed to bioaccumulation intracellularly. Meanwhile, the bioremediation of Cd^2+^ was a slow rise process within 6 h; the removal rates were from 61.7 to 74.6% at the concentration range of 0.5–4 mg/L, which was mainly mediated by surface biosorption. Subsequently, the adsorption rate decreased and then rose slowly, followed by achieving a dynamic equilibrium between the interactions of metal ions and the metal-binding sites on the surface of cells. Therefore, it is speculated that both surface adsorption and intracellular bioaccumulation participate in the removal mechanism of Cu^2+^, Pb^2+^, and Cd^2+^. The overwhelming majority of the heavy metals were adsorbed to the cellular surface of *D. salina* before 6 h, while only a minor percentage of them were bioaccumulated intracellularly within 6–72 h. Similarly, Komjarova and Blust (2009) reported that two-thirds of Cd^2+^, Zn^2+^, and Ni^2+^ concentrations were adsorbed to the cell wall of *Pseudokirchneriella subcapitata* after 72 h of exposure, and only one-third was inside the cells, except over 80% of Cu^2+^ and Pb^2+^ were bioaccumulated in the cells. Elleuch et al. (2021b) reported that *D. salina* exhibited an outstanding removal capacity of Pb, with nearly 95%. Both surface bioadsorption (49.34%) and intracellular bioaccumulation (50.66%) mechanisms were equally involved in the uptake of Pb by *D. salina*, whereas most of Pb removed by diatom cells were through a surface bioadsorption mechanism (approximately 75%). Moreover, owing to the existence of carboxyl groups in algae, the solution pH plays a crucial role in the elimination of heavy metals. At low pH, the carboxyl groups will be protonated because of a large amount of H^+^, which impede the joint efficiency of heavy metal ions. When the pH is too high, the hydroxide complexes will be produced, which reduced the concentration of dissolved metal in the solution (Ramesh et al., 2023).

Additionally, FTIR spectroscopy can be performed to identify microalgae components (e.g., lipids, proteins, carbohydrates, and carotenoids) and further explore the mechanism of adsorption and degradation of environmental pollutants, owing to its distinctive advantages of simplicity, high efficiency, and non-destruction (Zhang et al., 2020; Liu et al., 2022). For instance, Pelusi et al. (2016) discovered a characteristic absorption peak due to the stretching vibration of -C=C group of alkenones at 962.5 cm^−1^ in *Emiliania huxleyi*. Similarly, due to these functional groups existed in cell surface of algae such as hydroxyl (-OH), carboxyl (-COOH), amino (-NH_2_), phosphorous (-P=O), and sulfur (-S=O) groups, the cations such as Cu^2+^, Mg^2+^, Na^+^, and Pb^2+^ in wastewater were initially attached to the functional groups and exchanged with each other, thereby exhibiting biosorption function (Ye et al., 2015; Zeraatkar et al., 2016; Cheng et al., 2021; González Fernández et al., 2023). In this study, the shifts in the absorption bands of hydroxyl (-OH), carboxyl (-COOH), aldehyde (-CHO), carboxyl (-C=O), amine (-NH), amino (-NH_2_), phosphorous (-P=O), and sulfur (-S=O) groups were observed before and after Cu^2+^, Pb^2+^, or Cd^2+^ exposure using FT-IR spectroscopy. These functional groups may enhance the adsorption efficiency of Cu, Pb, and Cd ions through electrostatic adsorption or ion-exchange. Similarly, Elleuch et al. (2021b) claimed that these functional groups, including hydroxyl, carboxyl, amino, phosphate, and sulfhydryl groups, were also relevant to the biosorption of Zn, Pb, and Cr ions through the surface adsorption and intracellular bioaccumulation mechanism.

Despite several studies on the heavy metal adsorption by algae, most of the literature studies only pay attention to the removal capacity of algae on single metal under laboratory-controlled conditions. In the realm of practical and environmental matrices, the utilization of algae in the purification of waste streams containing multiple heavy metals continues to be an intractable problem. The coexisting metals in the environment often compete with the targeted metals for metal binding sites, resulting in a reduction in the removal capacity of the targeted metals (Yang et al., 2015). The competition highlights the intricate balance within the biosorption process and underscores the necessary strategies for enhancing the selectivity and efficiency of biosorption, thereby ensuring that the targeted metal ions are preferentially adsorbed. Recently, researchers have endeavored to develop novel algal biosorbents through molecular, chemical, and extraction approaches and nanoparticle (NP) synthesis techniques to modify algae, aiming to enhance adsorption capacities and elucidate the underlying mechanisms (Cheng et al., 2019). For instance, a cell-surface display technology by engineering the cell surface has been developed to overexpress the metalloregulatory proteins and molecules including metallothioneins (MTs), phytochelatins (PCs), glutathione (GSH), and proline; algae or transgenic algae can effectively absorb more targeted metal ions, showcasing a tailored and efficient approach to environmental remediation (Bae et al., 2002; Cobbett and Goldsbrough, 2002; Espinoza et al., 2021). To date, the most successful genetic transformation system comes from the green alga *Chlamydomonas reinhardtii,* attributing to the commercial applications of both nuclear and chloroplast transformation system. The self-flocculation property of microalgae significantly amplifies the potential of cell-surface engineering, rendering microalgae an economic and efficient biosorbent for the recovery of precious metals, such as gold (Li and Tao, 2015; Shen et al., 2017; Wang et al., 2023). It can be concluded that the technique established with *C. reinhardtii* could potentially be applied to other algal species.

Additionally, during the biosorption process, the cell wall of algae stands as the first obstacle against heavy metals, and its components can determine the sequestration mechanism of heavy metals. The diverse functional groups inherent in polysaccharides and lipids are pivotal in enhancing the adsorptive capability of algae, including carboxyl, hydroxyl, phosphate, sulfate, fatty acid, disulfide, amino, and sulfhydryl groups (Abdel-Aty et al., 2013; Xu et al., 2022). Recently, alkali and acid modifications have been widely employed to boost the biosorption potential of biological materials. Using pretreatment with NaOH solution, the negative charges on the surface of algal cells are augmented, and hydrolysis reactions lead to the production of more hydroxyl and carboxylic groups. Consequently, the biomass of algae experiences a significant enhancement in its metal-binding capacity. As reported by Bulgariu and Bulgariu (2014), the pretreatment with 0.6 mol/L of NaOH enhanced the biosorption capacity of *Ulva lactuca* by 11.75, 60.64, and 62.53% for Pb (II), Zn (II), and Co (II), respectively. Moreover, acid treatment leads to carbonyl stretching of carboxylic acids and protonation of functional groups, exposing more adsorption sites to enhance high affinity for targeted heavy metals. Interestingly, Yacou et al. (2018) reported that *Turbinaria turbinate* was subjected to H_2_SO_4_ and HCl; the adsorption capacities for Cr (VI) were enhanced by 16.8 and 246%, respectively. Taken together, these techniques and modifications not only augment the surface area available for metal binding but also generate new binding sites, thereby facilitating a stronger interaction with metal ions. This convergence of chemical and structural alterations converts the algal cells into proficient entities for metal sequestration, promising a cleaner and healthier ecosystem.

So far, the green alga *Dunaliella salina* has aroused a wide concern attributing to its peculiar structure and physiological characteristics. *D. salina* is a photoautotrophic organism which can utilize light to synthesize organic substance. It is noteworthy that *D. salina* can produce multiple compounds of industrial interest, such as carotenoids, glycerol, lipids, proteins, and pharmaceuticals (Cho et al., 2016; Pourkarimi et al., 2020). Additionally, *D. salina* can survive and reproduce under extreme conditions of high salinity, high temperature, and pressure due to its special structure and physiological characteristics. The salt-tolerant mechanism may be closely associated with the regulation of osmotic pressure, ion channels, metabolic pathway, and antioxidant system (de Souza Celente et al., 2024). Most importantly, the potential of *D. salina* extends far beyond the generation of these valuable bioproducts but also a promising candidate for a cost-effective approach for wastewater treatment. Multiple findings have revealed that *D. salina* exhibits a strong adsorption capacity for heavy metals, mainly attributing to its peculiar structure and composition of the cell wall. Despite *D. salina* lacks a rigid cell wall, it possesses a pericellular matrix composed of proteins and peptidoglycan which are linked alternately by N-acetylglucosamine and N-acetylmuramic acid. The special structure of *D. salina* containing various functional groups or metal-binding sites, including hydroxyl, carboxyl, amino, phosphate, and sulfate groups, as well as amine, alkene, alkyl halide, and alcohol moieties, may contribute significantly to the adsorption process of metal ions (Romera et al., 2007; Polle et al., 2020; Elleuch et al., 2021b). For instance, Kwon et al. (2017) claimed that *D. salina* exhibited outstanding bioadsorption for Zn, followed by Cu, Co, and Cd, which may be associated with the content of extracellular polymeric substances (EPS). Due to the absence of cell wall in *D. salina*, the production of EPS is likely triggered as a response to saline stress, forming a gelatinous polysaccharide layer around the cell and serving as a protective barrier against damage (Mishra and Jha, 2009; Johari et al., 2018; Sendra et al., 2018). Therefore, these peculiar characteristics make *D. salina* an important model organism for exploring the extreme environmental biology and heavy metal control. It is noteworthy that *D. salina* can be subjected to the industrial wastewater or effluent with the dual stress of heavy metals and salinity, and the nutrients in wastewater can facilitate the growth of *D. salina* and bolster the yield of high-value bioproducts that transfer the money-consuming wastewater treatment into a profitable rewarding venture. *D. salina* has a potential to be a superior, cost-effective biosorbent in the future.

Nevertheless, employing *D. salina* for wastewater treatment remains a challenge. One plausible explanation is that the application of microalgae in treating wastewater is predominantly confined to laboratory settings and lacks a comprehensive research on the feasibility, especially to yield relevant bioproducts concurrently (Kalra et al., 2021). On the other hand, despite the nutrients in wastewater can facilitate the growth of *D. salina* and bolster the yield of high-value bioproducts, the feasibility and applicability of using *D. salina* as a food source may be compromised due to the wastewater source, necessitating careful selection to ensure safety and efficacy. Finally, the utilization of *D. salina* in wastewater treatment may be hindered by the unpredictable variability of wastewater components, the continuous investment, and the fluctuating yield of biomass (Zhang et al., 2022b).

To overcome the limitations and broaden the application spectrum of *D. salina,* genetic engineering, chemical modification, and nanoparticle (NP) approaches have been arousing attention as a promising solution to improve the efficacy and selectivity of heavy metal biosorption (Feng et al., 2020). Moreover, to surmount the unfavorable conditions, an alternative strategy is to use non-viable cells to capture nutrients. Dineshkumar et al. (2024) identified *D. salina* as a superior, cost-effective biosorbent that was alternative to recover Pb from industrial effluent, which was associated with the presence of various functional groups, such as amine, alkene, alkyl halide, and alcohol moieties, participating in the adsorption process. Particularly, owing to the overwhelming capability of surviving under extreme circumstances of high salinity, temperature, and pressures, *D. salina* can be utilized to treat the wastewater with high salinity and heavy metals or cultivate in the wastewater, yielding valuable bioproducts, such as lipids, carotenoids, and glycerol. Therefore, due to the outstanding enrichment capacity, easy desorption and self-flocculation for heavy metals, *D. salina,* have been considered as a promising and environmental-friendly bio-adsorbent in industrial and environmental settings, facilitating the regeneration of bioproducts that transfer the money-consuming wastewater treatment into a profitable rewarding venture.

## 5 Conclusion

Microalgae possess a remarkable ability to adsorb heavy metals from aqueous solutions, primarily owing to their vast surface area and strong binding affinity, which enables them to serve as a promising solution for environmental remediation. Our research assessed the tolerant and removal capacity of *D. salina* toward copper, lead, and cadmium ion exposure at varying concentrations. Notably, Cu, Pb, and Cd ions remarkably restrained the growth and the synthesis of photosynthetic pigments, polysaccharides, and protein of *D. salina* under acute toxicity conditions. Under certain circumstances, heavy metal stress could stimulate the activities of antioxidant enzymes such as SOD and CAT, to defend the oxidative damage. When the concentration of heavy metals exceed the tolerance capacity of algal cells, the antioxidant enzyme activities are destroyed along with MDA accumulation, leading to the toxicity of heavy metals on *D. salina.* Correlationship and PCA analysis demonstrated that the variations of biomolecules could be used as a biomarker to indicate the toxic effects of heavy metals on aquatic algae. Additionally, *D. salina* was proven to be good biosorbents for Cu, Pb, and Cd ions; the order of adsorption capacity of *D. salina* for the three heavy metals is as follows: Cu^2+^ > Pb^2+^ > Cd^2+^. BBD design discovered that the optimal pH for maximal removal rate was 5–6 and the optimum adsorption duration was 6 h at a temperature range of 20–30°C. In addition, both extracellular adsorption and intracellular bioaccumulation are involved in the removal mechanism of Cu, Pb, and Cd ions with *D. salina*. Furthermore, these functional groups such as-OH, -COOH, -NH, -NH_2_, -P=O, and-S=O are vital and necessary for the removal of Cu^2+^, Pb^2+^, and Cd^2+^ by *D. salina*, which participate in algal biosorption by ion-exchange and surface complexation. These findings indicate that *D. salina* can be considered to be an excellent bio-adsorbent to remove heavy metals, which lays a theoretical foundation and potential value for dislodging heavy metal ions from the wastewater. In the future, the bioremediation mechanism of heavy metals by *Dunaliella salina* needs to be further exploited.

## Data availability statement

The original contributions presented in the study are included in the article/supplementary material, further inquiries can be directed to the corresponding author/s.

## Author contributions

MG: Data curation, Formal analysis, Visualization, Writing – original draft, Writing – review & editing. NL: Funding acquisition, Project administration, Supervision, Validation, Writing – review & editing. HT: Formal analysis, Investigation, Writing – original draft. CG: Data curation, Investigation, Resources, Writing – original draft.

QW: Investigation, Resources, Visualization, Writing – original draft.
